# Automated liquid handling robot for rapid lateral flow assay development

**DOI:** 10.1007/s00216-022-03897-9

**Published:** 2022-01-29

**Authors:** Caitlin E. Anderson, Toan Huynh, David J. Gasperino, Luis F. Alonzo, Jason L. Cantera, Stephen P. Harston, Helen V. Hsieh, Rosemichelle Marzan, Shawn K. McGuire, John R. Williford, Ciela I. Oncina, Veronika A. Glukhova, Joshua D. Bishop, David M. Cate, Benjamin D. Grant, Kevin P. Nichols, Bernhard H. Weigl

**Affiliations:** 1 Global Health Labs, Bellevue, WA USA; 2 Center for In Vitro Diagnostics, Intellectual Ventures, Bellevue, WA USA; 3 PATH, Seattle, WA USA

**Keywords:** Lateral flow assay, Optimization, High-throughput screening

## Abstract

The lateral flow assay (LFA) is one of the most popular technologies on the point-of-care diagnostics market due to its low cost and ease of use, with applications ranging from pregnancy to environmental toxins to infectious disease. While the use of these tests is relatively straightforward, significant development time and effort are required to create tests that are both sensitive and specific. Workflows to guide the LFA development process exist but moving from target selection to an LFA that is ready for field testing can be labor intensive, resource heavy, and time consuming. To reduce the cost and the duration of the LFA development process, we introduce a novel development platform centered on the flexibility, speed, and throughput of an automated robotic liquid handling system. The system comprises LFA-specific hardware and software that enable large optimization experiments with discrete and continuous variables such as antibody pair selection or reagent concentration. Initial validation of the platform was demonstrated during development of a malaria LFA but was readily expanded to encompass development of SARS-CoV-2 and *Mycobacterium tuberculosis* LFAs. The validity of the platform, where optimization experiments are run directly on LFAs rather than in solution, was based on a direct comparison between the robotic system and a more traditional ELISA-like method. By minimizing hands-on time, maximizing experiment size, and enabling improved reproducibility, the robotic system improved the quality and quantity of LFA assay development efforts.

**Graphical abstract:**

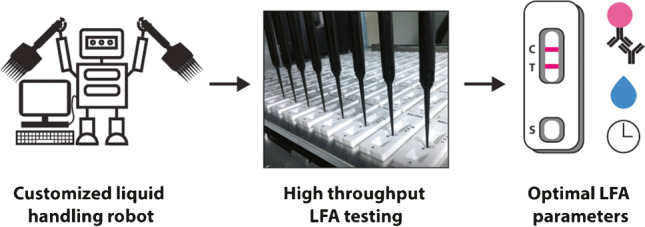

**Supplementary Information:**

The online version contains supplementary material available at 10.1007/s00216-022-03897-9.

## Introduction

The lateral flow assay (LFA) is a diagnostic technology that has enabled widespread point-of-care (POC) testing with wide-ranging applications. These applications include the detection of the pregnancy hormone human chorionic gonadotropin (HCG), environmental toxins, and a wide array of infectious diseases such as influenza, dengue, and malaria, to name a few [1–4]. The ubiquitous use of this technology is primarily due to its low cost and ease of use, without the need for refrigeration, electricity, or highly trained personnel [4].

In a typical LFA, reagents are deposited in different zones, such as the test membrane and conjugate pad, each of which is made of porous materials such as nitrocellulose, cellulose, glass fiber, or polymer fiber [5, 6]. The sample (e.g., blood, urine) and other liquids (e.g., running buffer) are applied directly onto the LFA by the user. Due to capillary action, the sample and any subsequent buffers will flow downstream into the test region without the need for additional pumping or pipetting steps [7]. The most popular LFA application is the immunoassay, in which immunocomplexes involving targets, antibodies, and reporter particles are formed at the test line to enable visual or fluorescence readout [8].

**Fig. 1:**
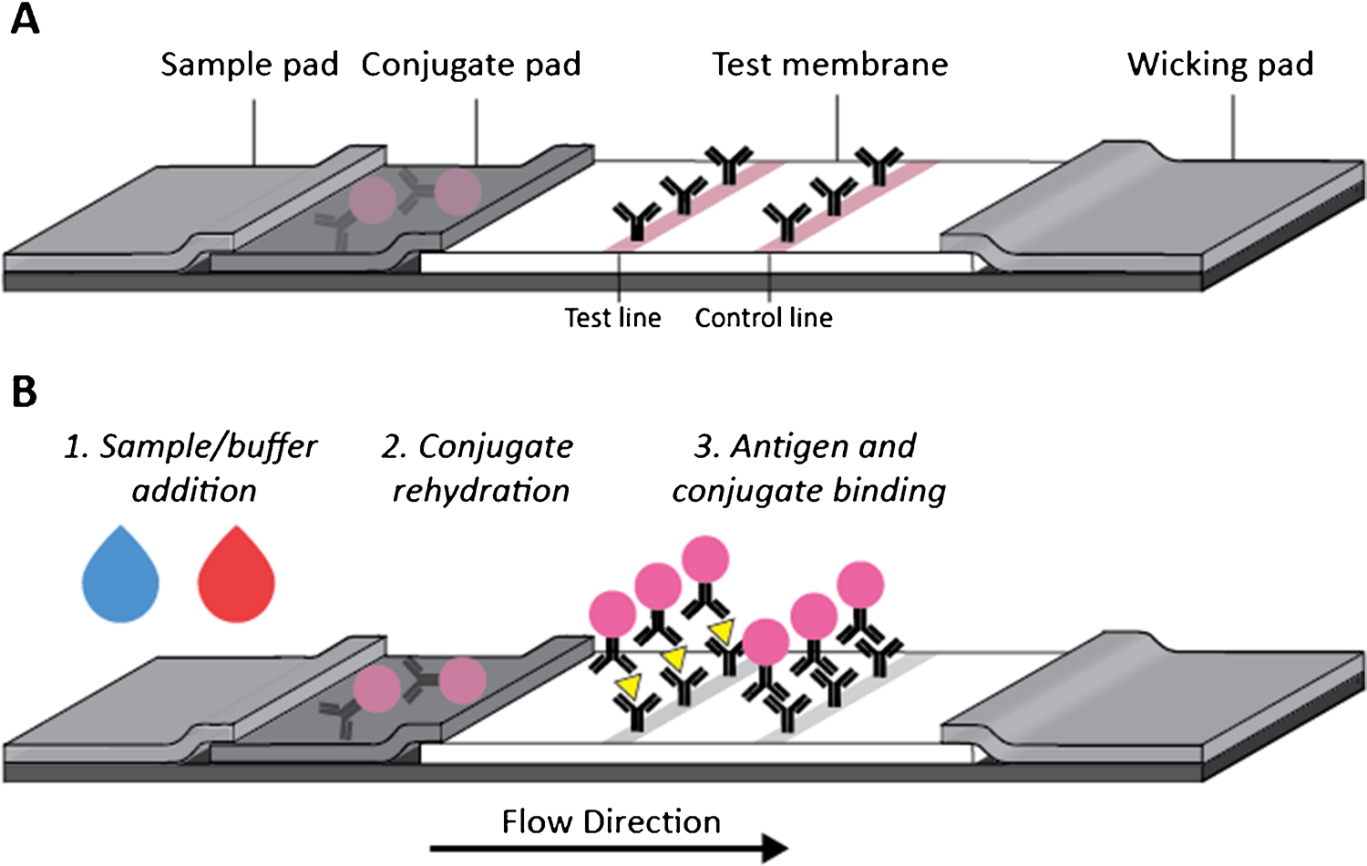
Schematic of a classic LFA, made from a combination of porous materials such as nitrocellulose, glass fiber, and cellulose (**A**). A traditional LFA involves the addition of a sample and/or buffer solution, which then flows downstream (towards the wicking pad) where it rehydrates the antibody conjugates (**B**). As the reagent solution continues to flow, through capillary action, the antigen and conjugate complexes will bind at the test line and conjugates bind at the control line. Some of the variables that are commonly tuned during the assay development process, can include (1) LFA strip material composition (2) material dimensions and placement, and (3) running conditions.

The development and optimization of LFAs remain a largely empirical and time-consuming process. A vast majority of the development and optimization occur in an entirely manual fashion. There is a need to more efficiently and rapidly develop LFAs due to increasing demands on performance [9] and from recent disease outbreaks [10–15]. LFA development involves many variables and typically goes through many iterations, [16] resulting in a lengthy and laborious process to find the optimal design. These variables, some of which are shown in Fig. 1, include the choice of materials, reagents, reagent concentrations, buffer volumes, and timing and order of reagent additions. To illustrate the impact of these variables on LFA development, the selection of reagents from between ten capture and ten detection antibodies and the testing at five concentrations each leads to a minimum of 2500 conditions to evaluate. The number of variables, and the combinatorial complexity of testing them, supports the argument for an improved, more automated system for LFA development and optimization.

The goal for automation of common laboratory practices is to streamline laboratory work while minimizing the amount of hands-on time required of trained personnel. The automated liquid handling robot, specifically, is a robotic system that has been demonstrated to improve laboratory processes by automating the pipetting component of a range of laboratory methods. The impact of these robots has generally been increased reproducibility, decreased cost, and increased overall efficiency [17–21]. Initially demonstrated to automate the highly repetitive enzyme-linked immunosorbent assay (ELISA), automated liquid handlers have since been used to automate numerous types of processes, such as nucleic acid extraction, purification, or assembly; cell culture; drug discovery; and materials discovery [18, 22–25]. By using an automated liquid handler to automate the preparation and running of LFAs, this work aims to improve the LFA development process by minimizing hands-on time while allowing for larger, faster, more streamlined experiments.

The integration of an automated liquid handling system with LFA development provides a unique capability previously impossible for traditional LFA development teams. Traditional LFA experiments are often limited in size and scope; consequently, larger optimization and screening efforts using higher throughput methods (e.g., ELISAs) are used for downselection before testing in the LFA format [26, 27]. However, conclusions from higher throughput methods do not necessarily translate to performance in an LFA, specifically due to the porous nature of the materials and continuous (but not necessarily homogeneous) flow of reagents [1]. In cases where the LFA development needs to occur quickly, such as pandemic response, there is a need for more rapid screening and prototyping in the LFA format. A system that allows for rapid, in situ LFA development and optimization provides a unique advantage in the LFA development space.

In this paper, we report on a new analytical tool for LFA development and optimization using robotic automation. The development workflow consists of five unique steps, experimental design, experimental setup, experimental validation, running of the experiment and data analysis, and the assay optimization process (Fig. S1). The platform provides significant benefits to assay development through in situ evaluations of LFA performance and combinatorial management of many variables unique to LFA development. First, we developed LFA-specific hardware and software for a commercial liquid handling robot and validated the resulting LFA development platform against traditional LFA development protocols. Next, we demonstrated the utility of the platform through development of LFAs for malaria and *Mycobacterium tuberculosis* antigens, with a specific focus on antibody selection and antibody concentration optimization. Lastly, we demonstrated the comparative benefit of the platform through direct comparison to an ELISA-like platform in a traditional antibody screen. This manuscript moves beyond previous publications and presentations by the inclusion of additional validation, links to all code and CAD files, and a direct comparison to more traditional antibody screening methods [26–28]. These demonstrations suggest this platform has the potential to revolutionize the LFA development process by minimizing hands-on time, maximizing experiment size, increasing reproducibility, and improving the management of experimental complexity.

## Materials and methods

### Hardware

**Fig. 2:**
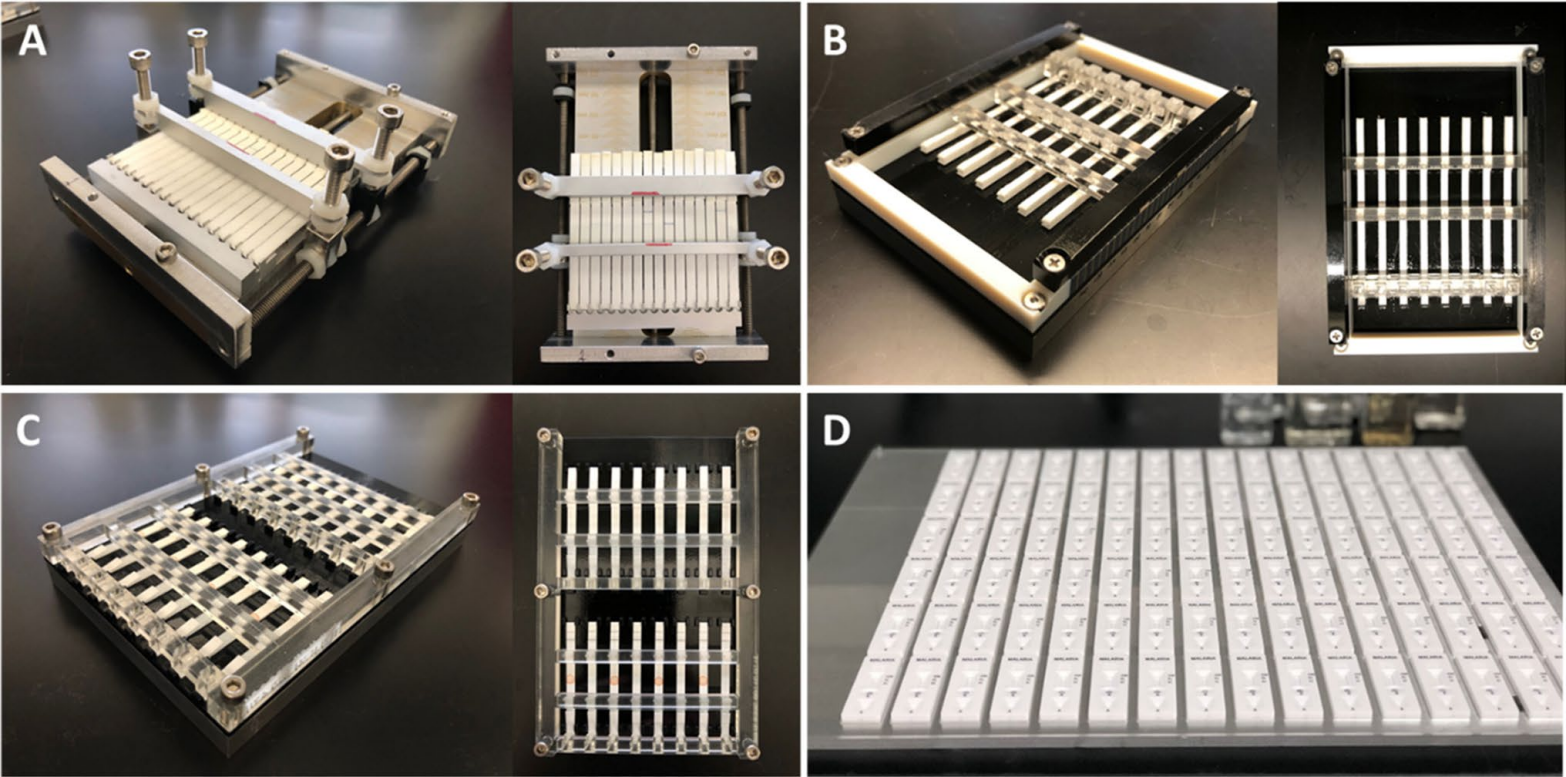
Images of the hardware components developed for the liquid handling robot, in the order of the most degrees of freedom (**A**) to fewest (**D**). The first three are strip holders that can each fit up to 16 strips, where (**A)** has the ability to vary the location and height for every well and pinch point manually, (**B)** fits together like Lego® pieces where all location and heights can be implemented with the library of pieces, and (**C)** a fixed system where each 3D-printed piece corresponds to one LFA design. The last piece of hardware (**D**) consists of machined and laser cut parts that hold 96 cassettes at a time. More information about each of these LFA holders can be found in Fig. S3–S6

The liquid handling robot (Hamilton STAR, Hamilton Company, Reno, NV, USA) was built with eight channels for parallelized pipetting and a camera for imaging (IDS UI-1460SE-C-H detector with a Tamron M118FM16 lens). Customized holders for the LFA test strips (strip holders) were designed and manufactured in-house, with pinch points to mimic supports otherwise provided by cassettes, or with slots large enough to accommodate cassettes around the LFA test strips. Version 1 strip holders were manufactured by machining aluminum and assembling with threaded rods (10-32) on both the top and bottom plates (Suppl Fig. S4). Versions 2 (Suppl Fig. S5) and 3 (Suppl Fig. S6) strip holders were manufactured using a 3D printer (Stratasys J750). Version 4 (Suppl Fig. S7) strip holders, which held cassettes rather than test strips, consisted of machined aluminum and laser-cut 1/16 in. acrylic. More detail about the hardware can be find in the Supplemental Information (Fig. 2).

### Software

Code was written to input experiment requirements and output lists of commands (denoted worklists) in Python 3.6. The inputs include protocols (with timing steps), choices of variables, and the layout of experimental conditions mapped onto the robot deck. This code also automatically splits the experiment into multiple worklists to accommodate the LFA capacity of the robot. The worklists are used as inputs for a master method written in the robot’s method editor, which converts the worklists into specific commands to send to the robot to execute.

Image analysis code was written and executed in Python 3.6. The code automatically identifies the region around the test line, performs background subtraction, and reports the height of the peak width-averaged pixel intensity of the test line as the signal. More information about the software can be found in the Supplemental Material.

### Clinical Samples

Nasopharyngeal swab samples were purchased from Medix (Lombard, IL, USA). Six SARS-CoV-2 negative and six SARS-CoV-2 positive samples were deidentified and pooled into their respective groups (“negative” and “positive”). Whole blood was purchased from Biological Specialty (Colmar, PA, USA). All blood samples were collected in anticoagulation tubes containing K_3_EDTA. Lithium heparin plasma was purchased from ProMedDx (Norton, MA, USA). Whole blood and plasma samples were all tested after pooling. Negative samples were tested as is, while positive samples were spiked at a known concentration with recombinant antigen (malaria antigen 1 or malaria antigen 2).

### Lateral flow assay

The assays were performed on LFA designs following the same format as Fig. 1, with sample and conjugate pads (6613, Ahlstrom-Munksjö, Oyg, Finland), nitrocellulose (CN95, Sartorius, Göttingen, Germany), and a wicking pad (440, Ahlstrom-Munksjö). Strips were cut to 3.3-mm wide and placed in injection molded plastic LFA cassettes. Reagents on the nitrocellulose test strips—poly-streptavidin (Cat #10,120,050, Biotez, Berlin, DE) at the test line and either donkey anti-chicken antibody (Cat #703–005-155, Jackson ImmunoResearch, West Grove, Pennsylvania, USA) or recombinant Protein A/G (Cat #21,186, Thermo Fisher Scientific, Waltham, MA, USA) at the control line—were dispensed using a commercial dispenser (ZX1010, BioDot, Irvine, CA, USA). Strips were dried at 25 °C for 30 min before storage in a desiccator until use.

Detection antibodies were conjugated using EDC/NHS coupling to either 400-nm carboxylic blue latex beads (CAB400NM, Magsphere, Pasadena, CA, USA) or cellulose nanobeads (NanoAct BL1AA, Asahi Kasei, Tokyo, Japan) using previously published protocol [29]. Capture antibodies were biotinylated using NHS-dPeg12-biotin (Cat #10198, Quanta Biodesign, Plain City, OH, USA) at a molar ratio of 20:1 for the malaria and SARS-CoV-2 assays and 10:1 for *M. tuberculosis* (MTB) lipoarabinomannan (LAM). Both conjugates were diluted in a dilution buffer of 50 mM borate containing 1% casein, 10% sucrose, and 2% trehalose. LFAs were run by the addition of biotinylated antibody to the conjugate pad and latex conjugated antibody to the sample pad and allowed to dry at 25 °C for 15 min. The sample matrices tested were plasma, lysed whole blood, or nasopharyngeal swabs, all of which were collected with consent, and were applied directly onto the sample port. For all assays described in this paper, strips were imaged at either 30 or 35 min after the addition of sample. LFA test strips that were run by a human were read on a commercially available LED-based LFA reader (AX-2X-S, Axxin, Fairfield, VIC, Australia).

Capture and detection antibodies were used for three different pathogens throughout the course of this manuscript. For simplicity, each antibody follows the naming convention of letter-Ab-number, where the letter indicates the antibody’s target. C indicates SARS-CoV-2 as target, M indicates malaria parasite as target, and TB indicates TB LAM as target. For example, the antibodies tested for SARS-CoV-2 would be named CAb001 through CAb005. Each individual antibody can be used for either capture or detection, with corresponding modifications listed above.

### Liquid electrochemiluminescence-based immunoassay

This immunoassay screen used the U-PLEX development pack, 10-assay (Meso Scale Discovery (MSD), Rockville, MD) on the MSD electrochemiluminescence (ECL) platform. Per protocol, two aliquots of each antibody (1 mg/mL) were labelled with biotin (EZ-Link Sulfo-NHS-LC-Biotinylation Kit, ThermoFisher Scientific) for capture and SULFO-TAG (GOLD SULFO-TAG NHS-Ester, MSD, Rockville, MD, USA) for detection. Unbound biotin or SULFO-TAG was removed using Zeba spin desalting columns (ThermoFisher Scientific), and the incorporation ratio for each label was measured. Each of the biotinylated capture antibodies was then mixed with specific U-PLEX linkers. To prepare the capture antibody arrays, up to 10 antibody-linker conjugates were combined in U-PLEX stop solution at a concentration of 0.29 μg/mL per antibody, and 50 μL of this mixture was added to individual wells of the U-PLEX 10-Assay, 96-well SECTOR plate. The plates were incubated for 1 h with shaking at 500 rpm to allow self-assembly of the antibody array to the complimentary antibody linker-binding sites. The unbound material was removed by washing 3 times with 300 μL/well of 1 × phosphate buffered saline + 0.05% Tween 20 (PBS-T, pH 7.5) using a BioTek ELX405R microplate washer (BioTek Instruments Inc., Winooski, VT, USA).

The 5000 pg/mL solution of LAM was prepared in 1% BSA. In each well in the plate, 25 μL of Buffer 22 (MSD) was added before the addition of 25 μL of the LAM sample; 1% BSA was added in a control well for background. The plate was incubated with shaking for 1 h at room temperature, then washed 3 times with PBS-T. A total of 25 μL of 2 μg/mL SULFO-TAG detection antibody in Diluent 3 (MSD) was added to each well, and then incubated for 1 h with shaking. The plate was washed 3 times, and the wells filled with 150 μL of 2 × read buffer T (MSD), and then read by MESO QuickPlex SQ 120 plate reader (MSD). The ECL from each individual array spot was subsequently measured using the Discovery Workbench v4 software and was used to determine the signal-to-noise ratio (S/N) or signal-minus-noise (S–N) for each antibody pair.

## Results

### Hardware and software development for liquid handling robot

Automated liquid handlers have been used for a wide range of applications, including enzyme-linked immunosorbent assays, cell culture, and nucleic acid amplification [30–32]. Despite their broad applicability, the use of automated liquid handlers for LFA optimization has been limited due to the need for LFA-specific hardware and software. Not only are LFAs formatted as non-standard labware in manually operated cassettes, LFAs themselves are not standardized, but typically unique to a set of assay requirements or a commercialization entity [33, 34]. Customized variables that define the operable interfaces of LFAs include the size (height, length, width) of the cassette, the location and number of sample or buffer wells, and the location of the visual read window. For the purposes of this work, a single LFA format was used. Hardware that allows the flexibility to address different LFA interfaces and custom software to control the hardware are therefore required in an automated LFA development platform. We started with a commercially available liquid handling robot and modified it with a camera to acquire images and custom labware made from machined and 3D-printed parts to hold a relatively large number of LFA test strips (denoted strip holders) in addressable locations on the robot deck. A traditional LFA, consisting of a few different materials with overlapping regions, uses supports inside the cassette that press on either side of the LFA to maintain contact and consistent flow between the overlapping regions. To be consistent with traditional LFA development, we developed four types of LFA holders (Suppl. Fig. S3–7), each to be used for a different stage of LFA development. The first holder type (Suppl. Fig. S4) was designed to fit a wide range of LFA geometries and provides the capability to change most important parameters of the LFA cassettes (locations and heights of pinch points and wells) by manually adjusting parts of the holder assembly. The second holder type (Suppl. Fig. S5) allows a similar flexibility but uses primarily 3D-printed parts instead. While the degrees of freedom for the second holder are slightly less, and the bar (wells or pinch point) placement can be changed in increments of 0.5 mm, assembling the LFA holder is significantly more reproducible and no longer requires machined parts. Once the locations of pinch points and wells have been determined, the third LFA holder type can be used (Suppl. Fig. S6). This holder type consists of two 3D-printed pieces, one each for the top and bottom, to provide pinch points and wells. Finally, the fourth holder type (Suppl. Fig. S7) is the housing for individual cassettes in the forms closer to the final products. More detailed information about all four types of LFA holders can be found in the Supplemental Material.

**Fig. 3:**
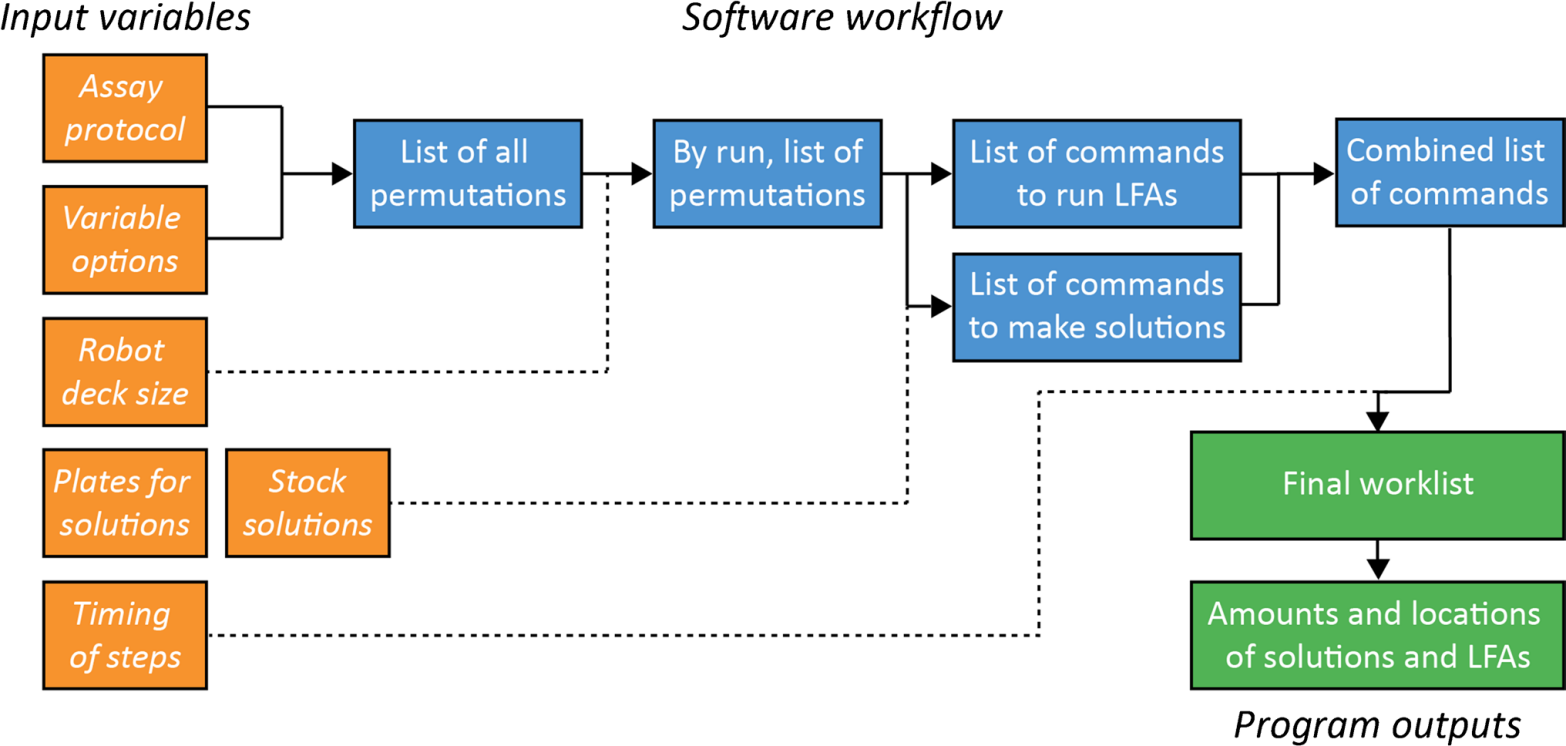
Diagram summarizing the worklist generator software developed for this work. More information, including instructions on how to use the worklist generator software, can be found in the Supplementary Information

The software comprises three modules, worklist generator, master Hamilton method, and image analyzer, all three of which are included in the Supplementary Material. Also included in the SI are results for the developed liquid classes for this work (Table S1–S3). The worklist generator (Fig. 3) takes inputs of assay protocol, experimental design (which variables to investigate), and robot settings. It determines the individual steps for the execution of assay permutations, and the preparation of the solutions from stock solutions. It then arranges the steps to satisfy timing requirements (e.g., time delay between reagent additions), groups the steps to take advantage of multi-channel pipetting, then writes a worklist in a format matching the requirements of the Hamilton master method into a.csv text file. The Hamilton master method was written on the Hamilton proprietary software platform to read the steps in the worklist (and the associated timing information), and to execute the protocol on the robot with the prescribed timing. In most cases with LFA runs, the execution also involves taking pictures, which are then analyzed by the image analyzer to quantify signals (spot or line intensities).

### Robot verification by comparison with human

**Fig. 4:**
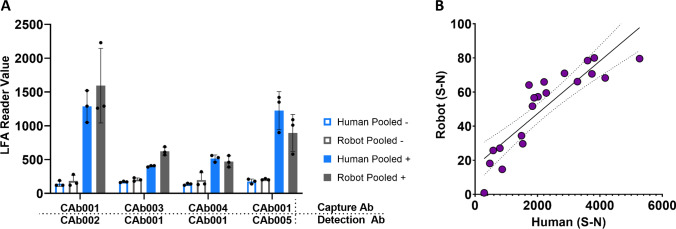
Verification of the performance of lateral flow assays performed on the robot, in comparison with those run by a human. (**A)** Plot comparing signal between LFAs run by the robot vs. a human operator for the SARS-CoV-2 nucleocapsid antigen. All strips were imaged using the commercially available AX-2X-S Axxin LFA reader. Antibody pairs run by both robot and human (*N* = 3). The matrix used in this experiment was pooled nasal swab samples. (**B)** Plot comparing performance of LFAs run and imaged using traditional LFA protocols (human) vs. the robotic liquid handling system (robot). Dotted line indicates 95% CI. The antigen tested was a marker for infection with *Plasmodium falciparum*. Antibody pairs run by the robot (*N* = 4) and by the human (*N* = 5). The matrix in this experiment was human normal pooled plasma

We verified the performance of this system by comparing results from robot-driven optimization of a model assay against corresponding results obtained by a human operator. The delivery of sample and reagents was tested first, on pooled nasal swab samples (with six samples in each of the SARS-CoV-2 negative and positive pools), using four different anti-nucleocapsid protein antibody pairs. Either a trained user or the liquid handling robot delivered sample and reagents, and all strips were read on an Axxin LFA reader. For all four antibody pairs tested, the variations between the two delivery methods (by human or by robot) were within the noise of a typical LFA (Fig. 4A), indicating negligible pipetting variation between the two methods.

Next, the imaging of LFAs was tested on the robot and in an Axxin LFA reader. The robot-run strips were imaged using the onboard camera and quantified using a custom Python script, while the human-run strips were imaged and quantified using an Axxin reader with Axxin image processing software. A direct comparison between these two imaging plus quantification methods demonstrated a linear correlation between the two methods (Suppl. Fig. S2).

For the final comparison, we tested 20 different antibody pairs to detect an antigen marker for *Plasmodium falciparum* (Fig. 4B). Building upon previous experimentation, each antibody pair was tested in LFAs run and imaged using traditional protocols (named “human”) and LFAs run and imaged using the robotic system (named “robot”). The conditions were matched except for three major differences: (1) membrane preparation, (2) sample and detection antibody mixing, and (3) strip readout. Membrane preparation in the “human” condition consisted of striping using a commercial liquid dispenser (Biodot), where the “robot” consisted of spotting using the Hamilton system. Sample and detection antibody mixing prior to delivery to the test strip occurred in the “human” condition, whereas detection antibody was dispensed first on the strip, followed by the sample, in the “robot” condition. Strip readout in the “human” condition took place using a commercially available LFA reader (Axxin), while the “robot” condition used the Hamilton integrated camera to take images analyzed using a custom Python script. For the twenty pairs run both by hand and on the robot, a high degree of correlation was found, with an *R* value of 0.8819. Considering the difference in material preparation and imaging, this data suggests that the robot can be used to replace a human with a high degree of confidence. The orders of the signals obtained by the robot and by the human are in agreement (Fig. 4A).

### Experiments with discrete variables

**Fig. 5:**
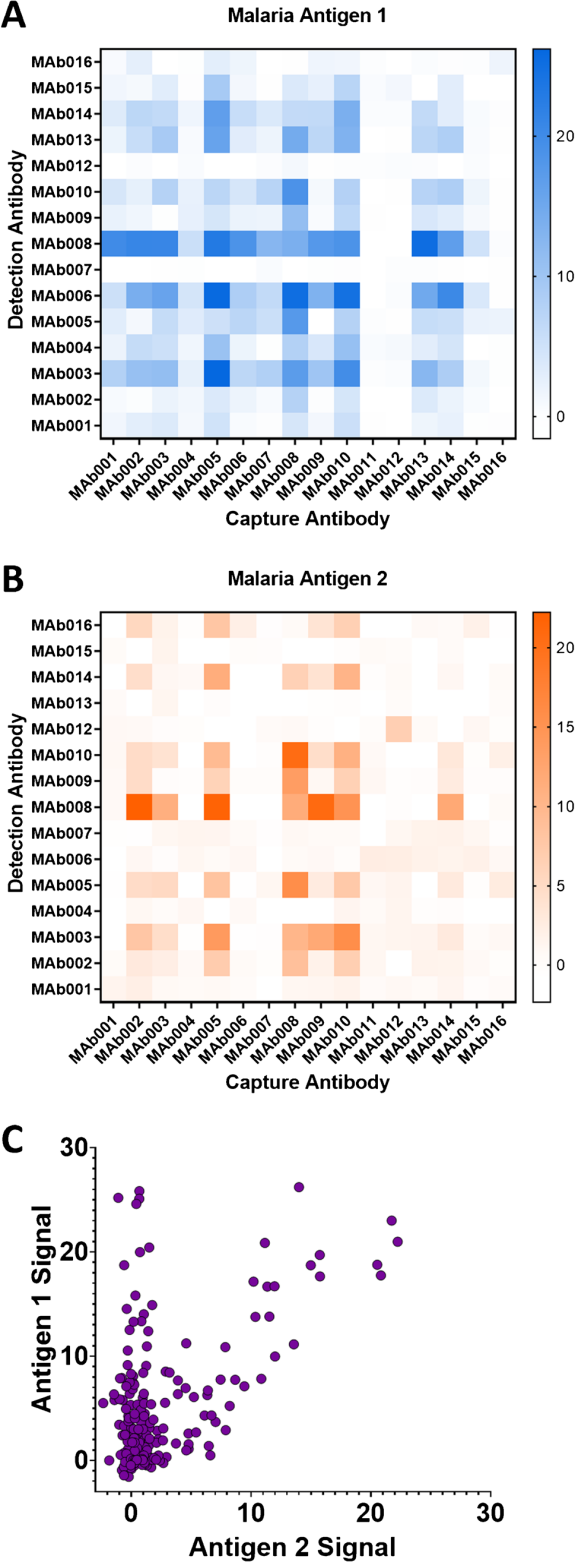
Screening of antibody pairs intended to detect an antigen from a *Plasmodium* species (ag1) but not a similar antigen from another *Plasmodium* species (ag2). The pooled negative plasma samples used for this work contain no native antigens, ag1, or ag2. Each of the capture antibody and the detection antibody can take 16 choices. Heatmaps of signals averaged over the duplicates for **A** antigen 1 (ag1) and **B** antigen 2 (ag2). **C** Scatterplot of blank-subtracted signals with ag1 versus blank-subtracted signals with ag2. The matrix used in this experiment was human normal pooled plasma

An important first step in LFA development is to select the binding reagents that will make up the assay stack. Most commonly, these binding reagents are antibodies, though they can include other proteins or nucleic acid sequences (aptamers) [35–37]. We demonstrated the capability of this system to screen discrete variables using the development of an assay for a marker of a *Plasmodium* species (ag1) that should not detect a marker from a different *Plasmodium* species (ag2) (Fig. 5). Discrete variables that are important to the LFA development process can include different antibodies, conjugation strategies, buffer systems, and LFA materials. In this example, we demonstrate the screening of different binding pairs to ag1, with a special interest in downselecting pairs that have non-specific binding to ag2.

The main goal of this experiment is to select promising capture-detection antibody pairs from an initially large set of options. In this experiment, there were 16 choices for each of the capture and detection antibodies (masked MAb001 – MAb016). To account for possible non-specific binding either to the matrix or to ag2, plasma, plasma spiked with ag1, and plasma spiked with ag2 were all included in the test matrix. With 2 technical replicates, the total number of LFA test strips was 16 × 16 × 3 × 2 = 1536. The experiment was split into 16 runs, which was executed over 3 workdays.

The signals from each antibody pair with both antigens, ag1 and ag2, were mapped as shown in Fig. 5A and B. Output signal was measured as the difference between each sample signal and the corresponding signals from the blank samples. Antibody pairs with the highest signal, as indicated in blue, for our antigen of interest were then further screened by the amount of cross-reactivity or non-specific binding found when the same antibody pairs were tested with ag2, as indicated in orange. A scatter plot (Fig. 5C) depicts the signal with ag2 plotted against the signal with ag1, indicating promising antibody pairs that were selective for ag1 (versus ag2). This method allows for much larger screens of binding pairs, quickly identifying promising antibody pairs for continued development. Among the highest performing pairs, four of the top seven had the same detection antibody and different capture antibodies. For this reason, one detection antibody was selected, alongside the three capture antibodies which showed the highest signal to ag1. Using traditional LFA development methods, this type of screen would have required significantly more time, both with respect to total run time as well as amount of hands-on time required by a trained laboratory personnel.

### Experiments with continuous variables

During the LFA development process, another major set of experiments required are those with continuous variables. These include testing of a range of concentrations of antibodies, buffer components, and assay timing. With these variables, the interest is to find either a minimum or a maximum response across the range of conditions tested. Using traditional methods, these optimization experiments are limited by the number of strips a trained laboratory personnel can run precisely during a given work period. Using an automated liquid handler, however, the size of the experiment is no longer limited in this manner. This allows for sufficient data to not only identify the best performer of concentrations tested, but really allows for a greater understanding of the experimental space being probed.

**Fig. 6:**
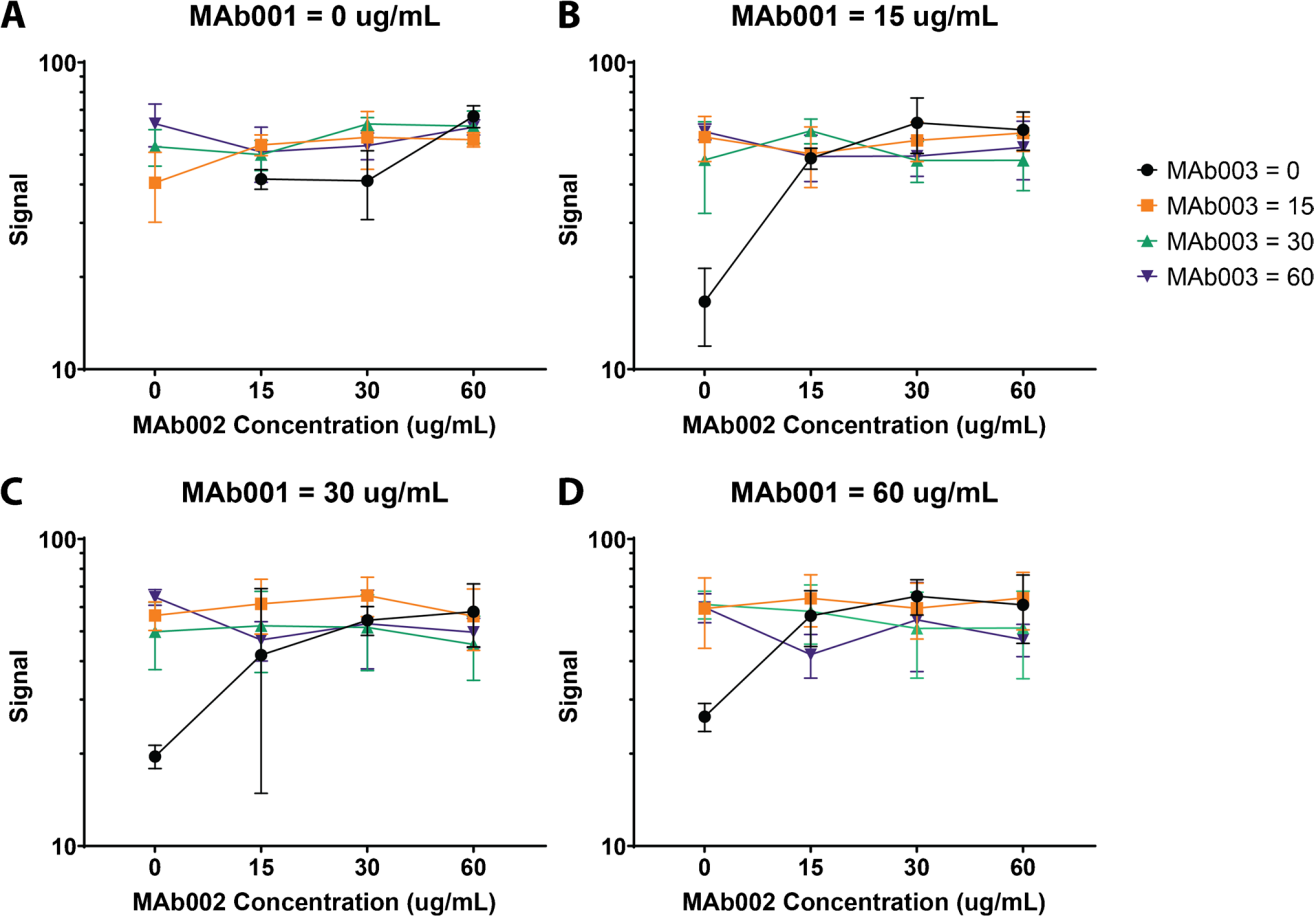
Example of an experiment involving continuous variables. The mixture of capture antibodies contained varied concentrations of each of 3 antibodies (masked as MAb001-MAb003 in this figure). Each plot represents signal while MAb001 is held constant, where MAb001 = 0 μg/mL in **A**, MAb001 = 15 μg/mL in **B**, MAb001 = 30 μg/mL in **C**, and MAb001 = 60 μg/mL in **D**. The x-axis represents MAb002 concentration, and MAb003 is depicted as individual data points on each plot. Each set of antibody concentrations was run in 4 replicates. The matrix in this experiment was lysed whole blood. The concentrations (ranging from 0 to 60) are in μg/mL

With the selection of the detection antibody (Fig. 5), the robotic liquid handling system was used to vary the concentrations of three candidate capture antibodies in their mixture (Fig. 6). Clinical performance of an LFA often benefits from having a combination of different antibodies for the same target, enabling broader specificity across a class of infections. Assays with 63 permutations of the concentrations of the capture antibodies were run with four technical replicates (Fig. 6). In total, 6 $3\times 4=252$ strips were run in half a day. The results indicate low signal across conditions where concentrations for MAb002 and MAb003 are both low. This assay shows little variability across many of the biotinylated antibody mixtures, with a slight decrease in signal when all three biotinylated antibodies are at the max concentration, suggesting the potential to move forward for optimization with multiple conjugate recipes. The use of a liquid handling system to determine the optimal number and concentration of antibodies conjugates allows for a larger sampling of the space than previously possible. The addition of multiple antigens, either of specific targets to detect or to avoid, can be easily introduced into this method to more accurately mimic the clinical needs of a given LFA.

### Comparison to traditional antibody screening methods

Traditional laboratory techniques including ELISA and ELISA-like platforms—the MSD ECL platform, for example—and label-free kinetic measurement platforms such as biolayer interferometry (BLI) or surface plasmon resonance (SPR) have historically been used for large screening efforts to feed into LFA development [38–41]. However, there are assumptions that are made during the screening and kinetic measurement process that may not apply to LFAs. For example, traditional kinetic measurement techniques operate under the assumptions that the reaction comes to steady state, that free diffusion is occurring, and that the reaction is reversible. Additionally, the microenvironments for binding in these techniques are typically homogeneous, with binding occurring on a well or other flat surface; unlike the porous, heterogeneous microenvironments for binding an LFA [42–46]. Taking these differences into consideration, we sought to directly compare screening results between the robotic LFA platform and the MSD ECL platform.

**Fig. 7:**
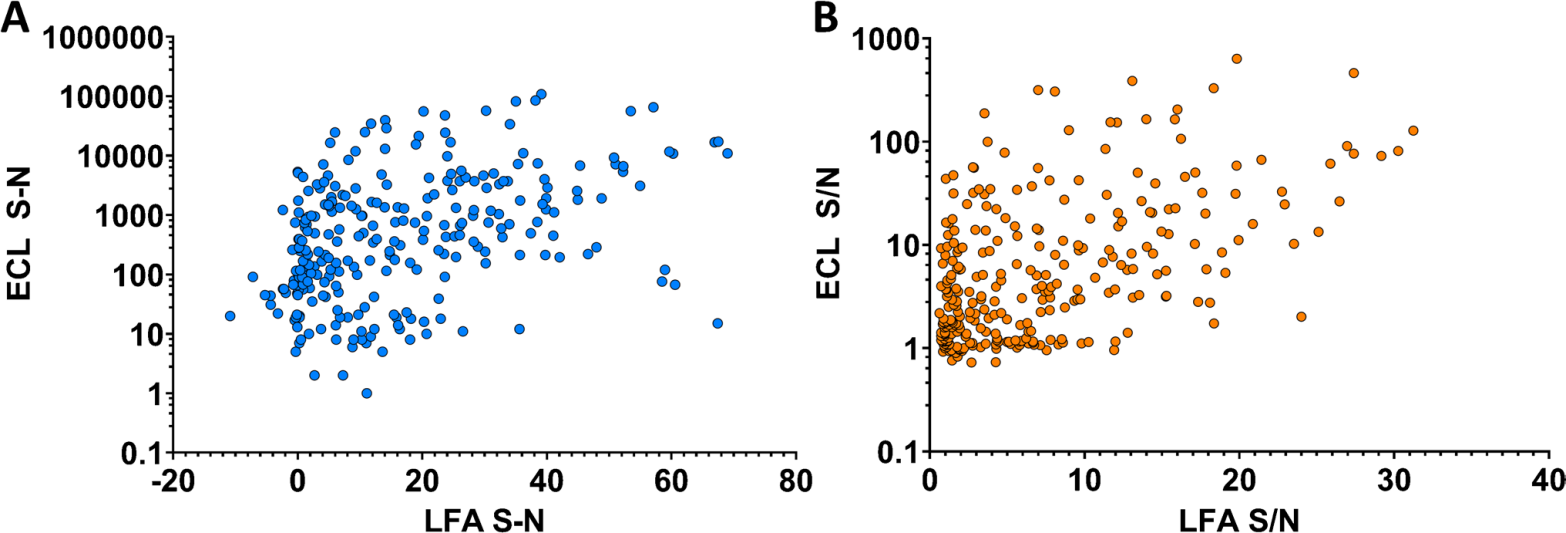
Scatter plot comparison between robotic LFA screening and ECL screening of LAM antibodies. 289 antibody pairs with *n* = 3 were compared for performance on each platform, looking at both S–N and S/N. Correlation values for both plots showed a weak positive correlation, with *R* values of 0.30 (*p* < 0.0001) and 0.36 (*p* < 0.0001) for S–N and S/N

To compare the robotic LFA platform to the MSD ECL platform, both assay stacks relied on streptavidin for capture, with a biotinylated capture antibody and labeled detector antibody, which was latex- or sulfo-tagged for the robotic LFA or MSD ECL platforms, respectively. Both assays were run with culture-derived LAM. The distributions of signal over noise (S/N) and signal minus noise (S–N) between the LFA and ECL platforms are shown in (Fig. 7A) and (Fig. 7B). There was a weak positive correlation found for both metrics, with *R* values of 0.36 (*p* < 0.0001) and 0.300 (*p* < 0.0001) for S/N and S–N, respectively.

Some discrepancy can be observed for certain antibodies and antibody pairs, most likely due to difference in assay timing, the size of conjugated particles, the methods for antibody conjugation, and other impacts of the local environment. With regard to timing, the LFA occurs under flow conditions where the capture and detector antibodies have limited time to bind while the ECL assay is incubated under shaking conditions for a significant amount of time, which can be 1 h or more. This discrepancy can bias away from antibodies with either a notably fast on rate or slow off rate, both of which can be particularly advantageous in the LFA format. Differences in the type of conjugation particle and the conjugation method used can also skew results. For example, additional testing with TAb005 found that the biotinylation ratio chosen for the initial screening effort inhibited performance (Fig. S9). These results highlight the need for platform-specific measurements, as there are the number of factors that can impact performance in an LFA that are not necessarily reflected in other platforms [44, 45, 47].

## Discussion

In this work, we have demonstrated the application of this platform to antibody pair and concentration selection, both of which are important steps in the LFA development workflow [16]. The goal of this work was to demonstrate the strength of this platform for LFA development efforts. Additional applications where this assay development platform can be particularly useful include everything from material choice, buffer volume, to assay run time testing, as shown in Fig. 1. The system was designed for colorimetric readout however; therefore, additional work would be required to outfit the system for detection of more sensitive readout methods (fluorescence, luminescence, electrochemical readout). From a practical perspective, the applications where this platform is the most useful are those in which the experiment is large, reproducibility is of concern, and/or when timelines are tight. It is important to note that while this platform automates some of the assay development process, the manufacturing process occurs separately. Experiments where this platform provides a distinct advantage are those where the manufacturing of LFA materials and antibody conjugates is streamlined such that the overall experimental timelines are reduced. Antibody pair selection is one such application where the capabilities provided by the automated system can allow for rapid screening efforts. 

The SARS-CoV-2 pandemic that began in late 2019 demonstrated the need for rapid diagnostic testing at the point of care, both for clinical response and epidemiological tracking of the virus. Companies around the world quickly began producing SARS-CoV-2 specific reagents in early 2020, including both antigens and antibodies, with little to no information about how well they would perform in an actual LFA. Using all commercially available SARS-CoV-2 antibodies, we created factorial matrices that allowed us to massively screen antibodies specific to the two main antigen targets, the spike and nucleocapsid protein. We used the automated liquid handling system to rapidly screen for over a thousand potential pairs of antibodies for spike and nucleocapsid antigen targets combined. More information about this screening effort can be found in the References Section [26, 27]. The rapid nature of this work would not have been possible without an automated lateral flow assay development system.

To our knowledge, this is the first implementation of an automated liquid handling system for LFA development. This system has the potential to streamline the LFA development process by increasing the potential experiment size while simultaneously decreasing the required hands-on time by trained personnel. Practically, such acceleration in LFA development can help deal with time-sensitive health events, such as disease outbreaks, when LFAs can be used for diagnostics in locations far away from central laboratories. Fundamentally, the wealth of data this system can provide can enable better understanding and modeling of LFAs.

## Conclusions

In this work, we described an integrated robotic system for LFA development, with signals comparable to those generated by a human through traditional laboratory practices. We demonstrated that the system performs experiments with both discrete and continuous variables, covering most types of assay development experiments required for LFAs. Additionally, this system allowed us to rapidly screen for the best performing antibodies for malaria parasite and *M. tuberculosis* LFAs in a rapid and reproducible manner. Not only were these experiments highly informative, but they also enabled reproducible results from experiments much larger than could traditionally be run on the bench.

The application of this system extends beyond the examples demonstrated in this work, which focused on traditional sandwich assays for direct antigen detection, to include other LFA designs and assay types. Not only does this system allow for larger experimentation with reduced hands-on time, but the application of an automated liquid handling system for the optimization of LFAs is also of particular interest for assay development related to time-sensitive events and/or resource-limited settings due to the potential reduction in overall development time and cost. This can have significant impact in the LFA development pipeline, a process that has remained mostly manual for decades.

## Supplementary Information

Below is the link to the electronic supplementary material.

Supplementary file1 (PDF 1122 KB)
