# Supplementary material for: Automated liquid handling robot for rapid lateral flow assay development
Source: Anal Bioanal Chem. 2022 Jan 29;414(8):2607–18. doi: 10.1007/s00216-022-03897-9 (PMC8799445; doi:10.1007/s00216-022-03897-9)
Supplement: Supplementary file 1 — Supplementary file1 (PDF 1122 KB) [file 216_2022_3897_MOESM1_ESM.pdf]

Supplemental Information:

Automated liquid handling robot for rapid lateral flow assay development

Caitlin E. Anderson[1*§], Toan Huynh[2§], David J. Gasperino[2], Luis Alonzo[1], Jason L. Cantera[1,3], Stephen P. Harston[2], Helen V. Hsieh[2], Rosemichelle Marzan[2], Shawn K. McGuire[2], John R. Williford[2], Ciela I. Oncina[2], Veronika A. Glukhova[1], Joshua D. Bishop[1], David M. Cate[1], Benjamin D. Grant[1], Kevin P. Nichols [1], Bernhard H. Weigl[1*]

[1]Global Health Labs, Bellevue, WA, USA

[2]Center for In Vitro Diagnostics, Intellectual Ventures, Bellevue, WA, USA

[3]PATH, Seattle, WA, USA

[*]caitlin.anderson@ghlabs.org, bernhard.weigl@ghlabs.org

[§]These authors contributed equally.

Table of Contents

A. Materials provided with manuscript 2

1. Hamilton Master Method 2
2. Hamilton Hardware definitions 3
3. Hamilton Liquid Class definitions 3
4. Python code to generate worklists 4
5. Python code to analyze images 4
6. Lateral flow assay (LFA) specific hardware 5

B. Additional application: nitrocellulose membrane spotting 8

A. Materials provided with manuscript

Alongside our manuscript, we have provided additional materials to enable replication of our work. This includes both software and hardware developed specifically for the application of lateral flow assay (LFA) development. All support files described below are available on GitHub (https://github.com/Robot-LFA). The general workflow of steps during an experiment using this system starts from choosing variables to investigate and ends with result analysis (Figure S1). Along this workflow, there are 3 major pieces of software developed for this work, the Hamilton Master Method, the worklist generator, and the image analyzer. Information about each piece of code can be found in their respective sections below.

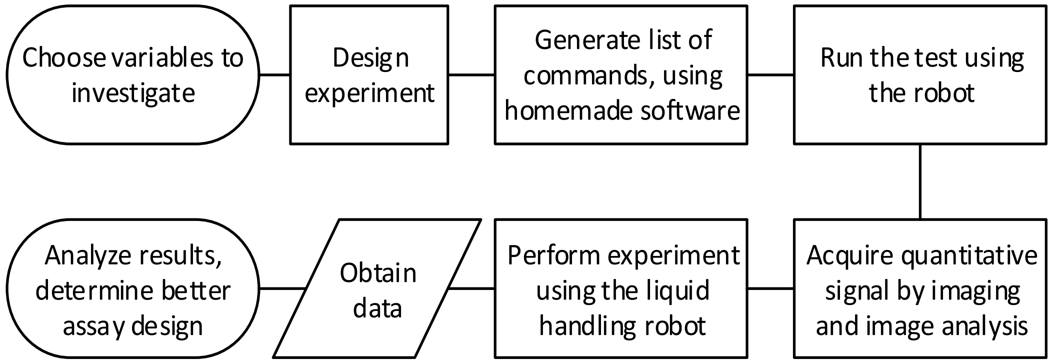

Figure S1. Diagram depicting the steps of the integrated robotic system in an experiment during assay development.

As an example workflow, at the beginning stages for LFA development one of the first steps is to select the antibody binding pair. The choice of capture and detection antibody would be selected as the variables to investigate for this experiment (Figure S1). During this process, the assay format (e.g. direct sandwich immunoassay, competitive immunoassay, or modified sandwich immunoassay with streptavidin capture) would be an important variable. In addition, at this stage the LFA geometry and specific materials to be used would need to be defined. The exact experimental details, including the specific antigen to be tested, number and types of pipetting steps, number of imaging steps, number of replicates, etc, would then be defined based on the constraints of that specific LFA. The LFA holder would also be chosen here, as the choice will depend on the availability. This is also the time to confirm that the appropriate hardware and liquid class definitions are selected.

From this information, the worklist to be input into the Hamilton Master Method would then be generated using the Python worklist generator code. We recommend running all new worklists in simulation mode before running the full experiment, alongside a small sample test where the method, imaging, and image analysis can be validated. Once any additional tweaks to the worklist have been made, the larger experiment can be performed. For many of our experiments, a single “experiment” could consist of an entire week’s worth of robot runs. The images acquired from the robot can then be analyzed using the Python image analysis software to quantify test line intensities, which are then used for result analysis and further experimentation. This is an example workflow for the use of this system, as you can image the number of variables that can be tested on a system such as this are many.

1. Hamilton Master Method

The **Hamilton master method** reads the worklist and executes the experiments with proper transfer sources and destinations, liquid classes, time delay, etc. as specified in the worklist. The worklist typically instructs the robot to acquire images as the results of the assays. The master method was written using the Hamilton Venus software and is included in the attached package (***ivl_master_method_robot_paper.pkg***).

Alongside the master method, for this system to work effectively we made (1) hardware definitions in the Hamilton software specific to the LFA hardware designed for this work (more detail about the hardware itself below), and (2) liquid class definitions for the liquids used for this work. More information about each of these is included below.

2. Hamilton Hardware definitions

Hardware definitions were designed specifically for the hardware used for this work. This was done within the Hamilton software, specifically for the “deck” where the LFAs are placed during each run. The hardware definitions are also included. For each individual machine, some optimization in house might be required.

3. Hamilton Liquid Class definitions

Liquid classes were designed specifically for the aqueous reagents used for this LFA, a running buffer containing detergent and whole blood. This was done using the Hamilton Liquid Verification Kit. Depending on the pipetting step, whether dispensing into a well or onto nitrocellulose, a different dispense method was required (Jet empty vs. Surface empty), therefore optimization took place for both. For blood, we chose not to use Jet empty to prevent the generation of aerosols. Below are tables that contain coefficient of variation (CV) for each volume as measured by the new liquid classes. These liquid classes are attached (***ivl_liquid_classes.mdb***), however due to the nature of liquid handling robots it is recommended to do optimization in house, as small variables in humidity and altitude can have a large impact on pipetting performance. Because of this, we recommend doing in house optimization of the liquid classes on your robotic set up before beginning any experimental work.

Table S1. Liquid class for the LFA running buffer using jet empty dispense. The average volume and CV are listed for each volume.

LFA running buffer - Jet empty dispense

50uL tip			300uL tip			1000uL tip		
Volume	Average Volume	CV	Volume	Average Volume	CV	Volume	Average Volume	CV
**1**	1	12.30%	10	9.9	1.50%	20	19.9	0.60%
**2.5**	2.6	10.80%	20	20.1	0.60%	50	50.1	0.60%
**5**	5.1	1.40%	30	29.9	0.50%	100	100	0.20%
**10**	10.1	1.00%	50	50	0.30%	200	200.4	0.10%
**20**	20.1	0.50%	100	99.8	0.20%	300	299.1	0.40%
**30**	29.9	0.90%	200	199.9	0.40%	500	500	0.40%
**50**	49.9	0.30%	300	300.2	0.10%	1000	999.3	0.20%

Table S2. Liquid class for the LFA running buffer using surface empty dispense. The average volume and CV are listed for each volume.

LFA running buffer – Surface empty dispense

50uL tip			300uL tip		
Volume	Average Volume	CV	Volume	Average Volume	CV
**1**	1	4.50%	10	10.1	2.10%
**2.5**	2.5	3.20%	20	19.9	1.30%

**5**	5	2.10%	30	30	0.50%
**10**	10	0.70%	50	49.9	0.30%
**20**	19.9	0.50%	100	100	0.20%
**30**	30.1	0.50%	200	200.2	0.50%
**50**	50	0.40%	300	300.1	0.10%

Table S3. Liquid class for the whole blood using jet surface dispense. The average volume and CV are listed for each volume.

Whole blood - Surface empty dispense

50uL tip			300uL tip		
**Volume**	**Average Volume**	**CV**	**Volume**	**Average Volume**	**CV**
**1**	1	4.60%	5	5	2.80%
**5**	4.9	1.90%	10	10.2	1.70%
**10**	9.9	1.50%	20	20.1	1.20%
**30**	30.1	0.50%	50	50	0.70%
**50**	49.9	0.40%	100	100	0.20%
			200	199.6	0.30%
			300	300.6	0.20%

4. Python code to generate worklists

The **worklist generator** inputs variables to investigate (which solutions, or dispense locations, or dispense volumes) and outputs worklists (list of commands for the robot). The code is in the attached file (***robot_worklist_generator.zip***). More information on how to run the code can be found in the included README.

5. Python code to analyze images

The **image analyzer** finds the regions of interest (signal lines or spots) on the resulting images and quantifies the corresponding signal intensities (***robot_image_analyzer.zip***). This code can be modified as required by the location of the test and control lines. One important validation step was that we compare the imaging and image analysis process between this new system and our “gold standard” LFA reader that we use for development. LFAs are most commonly read by eye, or with an LFA specific reader, though there has been a push in recent years to use cell phones to read LFAs. The Axxin reader is a commonly used instrument that is both CE approved and FDA ready, and has been used around the world for LFA readouts. Here, we compared the signal readout for strips with a range of antigen concentrations to determine whether the signal we read from the robot correlates with the reads from the Axxin. This data, as shown in Figure S6, shows a linear fit when plotting the Hamilton signal vs. the Axxin signal. This suggests that the signal analysis run on images acquired by the camera on the Hamilton STAR are sufficient for our purposes.

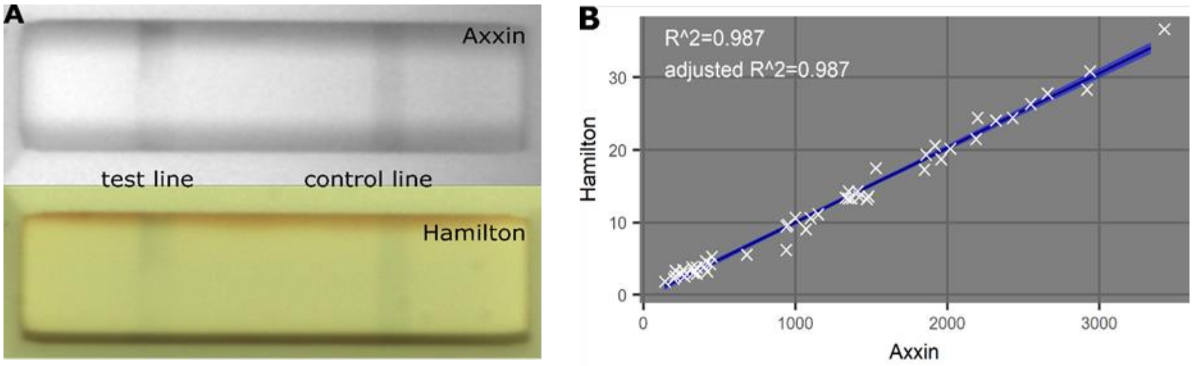

Figure S2. Correlation between signals obtained using a commercially available reader (Axxin) and the robotic system described here (Hamilton). A) Photographs of the same example LFA taken on the Axxin and on the Hamilton. B) Plot showing Hamilton signals versus Axxin signals corresponding to test lines on 48 LFAs. The LFA shown in A had Axxin signal of 1330 and Hamilton signal of 13.42. The linear fit yielded a slope of 0.0103 and an intercept of -0.254. The blue band indicates the 95% confidence interval of the linear fit.

6. Lateral flow assay (LFA) specific hardware

As described in the main text, we have developed four different hardware components to enable testing of LFAs on a Hamilton STAR robot. Computer aided design (CAD) files are included in the Supplemental Material (***robot_paper_CAD.zip***). The labware definitions are included in the master method package (***ivl_master_method_robot_paper.pkg***).

The first holder is a fully adjustable lateral flow assay holder, as shown in Figure S1. The parts were made by an in-house machine shop. The bottom base was made of stainless steel, and the other parts were made of aluminum. The threaded rod was made of stainless steel (10-32). The nuts for position adjustment were made of plastic.

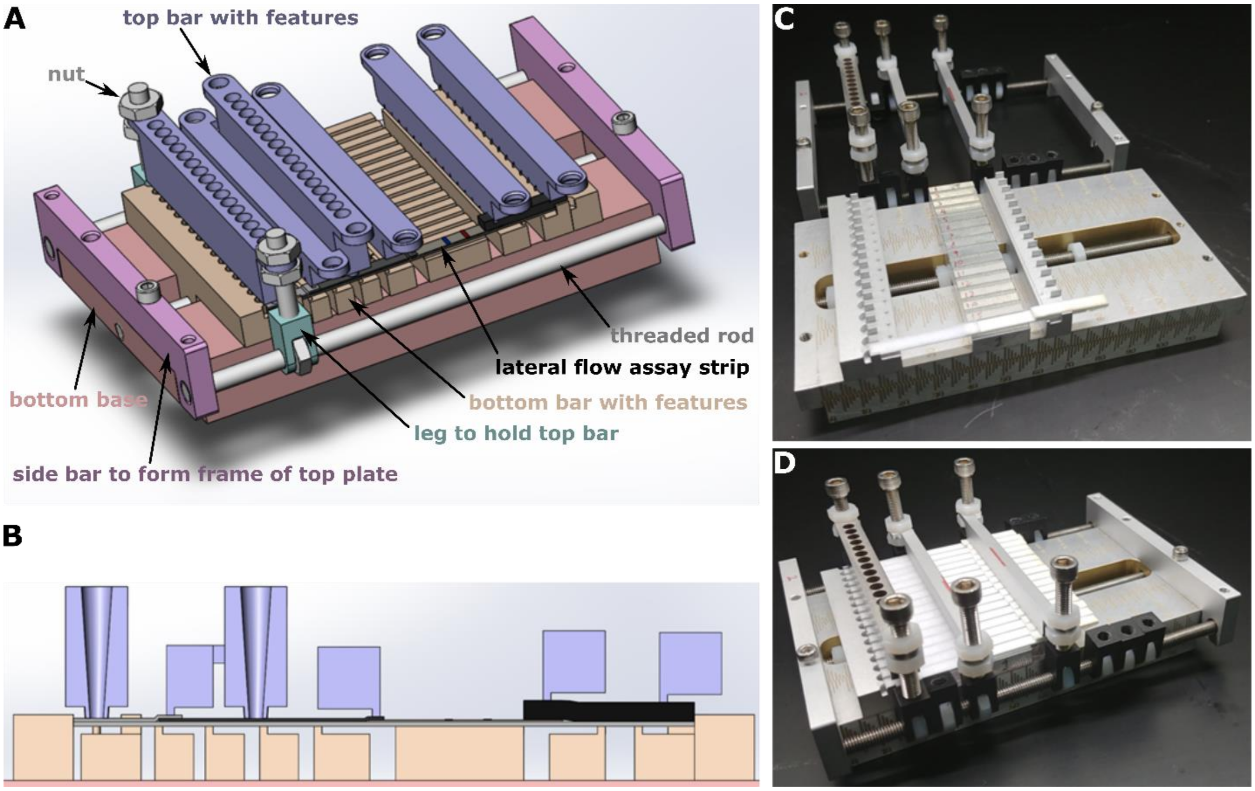

Figure S3. Adjustable lateral flow assay (LFA) strip array holder. A) Cartoon of an assembled LFA strip array holder. For clarity, only 1 LFA strip one 1 pair of side leg/rod/nut sets to connect the top bars to the top plate are shown. B) Cutout side view showing contacts between the LFA strip and features on the top and bottom plates. C) Photograph of a top plate and a bottom plate with 1 LFA strip. D) Photograph of an assembled holder with 16x1 LFA strips.

The second lateral flow assay holder was designed to substitute a degree of freedom for adjustment (height) with a set of pre-made 3D printed cross bars (Figure S4, Figure S5). The user cannot freely adjust the heights of pinch points and wells like with the first holder, but can freely choose different cross bars with different pinch point and well geometries. The cross bars and all other parts, besides the bottom base, were 3D printed using Vero inks on a polyjet printer (Stratasys J750). The aluminum bottom base (to correct for any warping of the 3D printed parts on top) was machined to have 2 sets of 3 threaded holes, located along the 2 long sides of the rectangle, which has the same footprint as the regular well plate. The bottom 3D-printed plate serves as the floor onto which LFAs are laid. The white frame provides teeth along the longer edges with rulers on the side. The clear cross bars containing pinch points or wells have teeth on the 2 ends to register with the teeth on the white frame. The black clamp bars are used to fix the cross bars to the white frame. The attached slide deck (Adjustable_Array_Strip_Cartridge_paper_SI.pdf) explains how this holder is used.

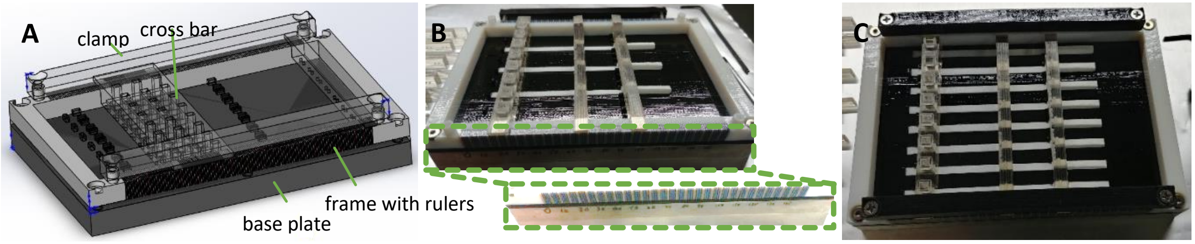

Figure S4. LFA strip array holder with laterally movable features (e.g. pinch points and wells. A) Cartoon of an assembled LFA strip array holder. B) Photograph of a partially assembled holder, without clamps. The inset shows the side ruler. C) Photograph a fully assembled holder. The base plate was made of aluminum. All other parts were 3D-printed (Vero ink, on Stratasys J750). Screw size: 6/32 x ½".

The third lateral flow assay holder is used when the locations of pinch points, wells, etc, have been determined using the first or the second holder (Figure S5). It was also made using primarily 3D printed parts (Vero ink on Stratasys J750). This holder uses the same aluminum base as the one in the second holder. The black 3D printed bottom plate also serves as the floor onto which LFAs are laid. It provides bars to serve as pinch points and rectangular features to guide the LFA into a specific location. The monolithic 3D printed top plate provides pinch points and wells, with predetermined locations specified in the CAD.

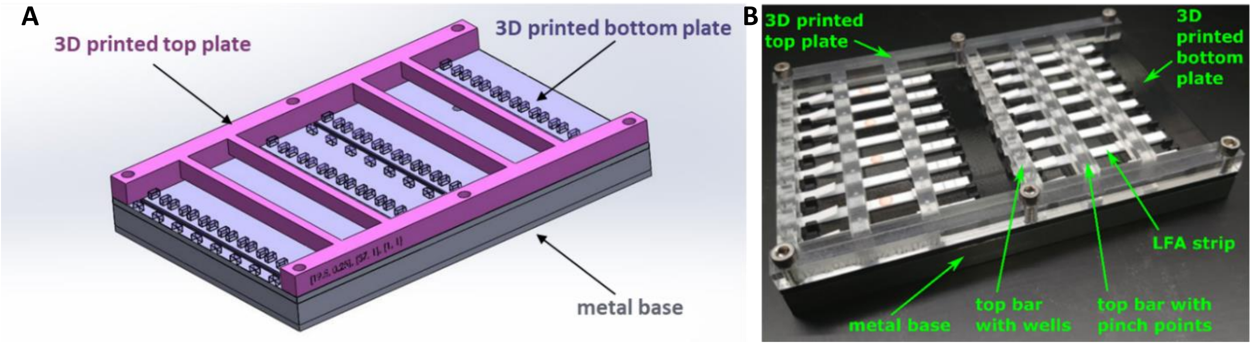

Figure S5. Modeling photo (A) and photograph (B) of an assembled printable lateral flow assay (LFA) strip array holder. The top plate was printed using a clear resin. The bottom plate was printed in black. The LFA strips are arranged in an 8x2 array. Screw size: 6/32 x ½".

The last piece of hardware developed for this work was designed to hold individual LFA cassettes with defined spacing, allowing for precise delivery across a larger number of LFA devices. The cassettes are typical of the final or close to final design; they can even be commercial cassettes. This cassette holder consists of an ¼" thick aluminum sheet (40 by 48) that has two pin holes that guide the sheet into place. On top of the aluminum sheet is a piece of 1/16" clear acrylic (McMaster-Carr) that was laser cut (Universal Laser Systems ULTRA X6000) with space for 96 cassettes of a given size. The number and location of pipetting locations on this tray correspond to labware designed in the Hamilton software.

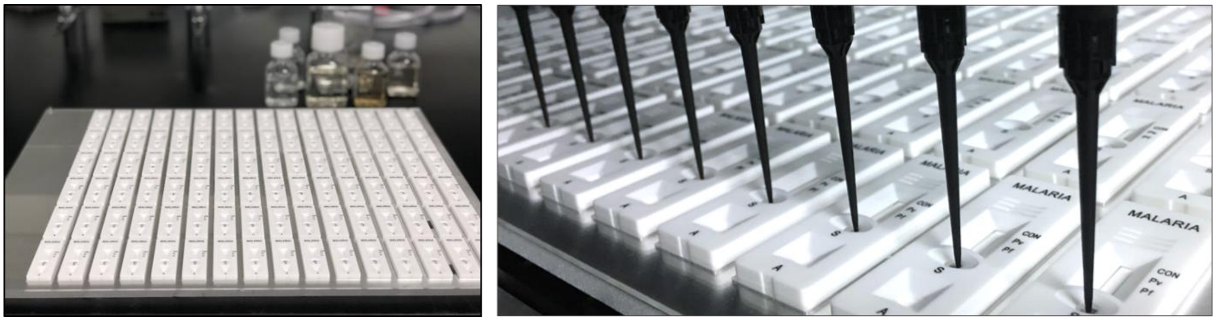

Figure S6. Photographs of a cassette holder designed for this work.

B. Additional application: nitrocellulose membrane spotting

In experiments comparing many different solutions of the capture reagent (e.g. the major capture molecule, the matrix, the concentration), it may be faster efficient to use the robot to spot the solutions, instead of striping multiple pieces of many pieces of nitrocellulose, which requires time for washing and loading between solutions. However, spotting solutions onto nitrocellulose is challenging because the material is fragile, and the volume is small (typically 1 µL per strip). We designed the hardware to be flat, tuned the liquid class (liquid class *ivl_tip50_spot_JetEmpty* in the attached *ivl_liquid_classes.mdb*), and adjusted the spotting height to achieve optimal spotting (1 mm above the nitrocellulose). To verify, we used the robot or a human operator to spot biotinylated mouse IgG (see protocol below), ran gold nanoparticle-streptavidin through for detection, and quantified the spot intensities (Table S4). We found that the coefficients of variation (CVs) of the robot were comparable to those of the human operator. operator (Figure S7, Table S5). This result indicates that the robot can spot reagents consistently on nitrocellulose and can help facilitate the screening many capture reagents.

Preparation of biotinylated IgG

- Biotinylation reagent stock: NHS-PEG$_{12}$-Biotin (Thermo 21312, lot SJ253341), 250 mM stock in DMSO (25 mg solid reagent in 82 µL of DMSO), stored at -20 °C.
- Mouse IgG (Equitech Bio SLM56-0100, lot 161201-0356): 10 mg/mL solution in PBS (100 mg in 10 mL of PBS), stored at 4 °C.
- Reaction: made a solution with the following components, mixed, then let react for 22 hours at 4 °C.
- Purification to remove unconjugated biotin:
    - Columns: Amicon columns (Millipore UFC50596 lot R8HA79021, 0.5 mL)
    - Used 2 columns. Split the reaction mixture evenly into the 2 columns
    - Centrifuged at 14000 rcf for 10 minutes, discard the low molecular weight fractions that passed through the column
    - Added 400 µL of PBS to each column, centrifuged at 14000 rcf for 10 minutes
    - Flipped the columns, centrifuged at 1000 rcf for 2 minutes to recover the high molecular weight fraction
    - Added 50 µL of PBS to each column to wash and combine with the primary fraction
- Estimated the concentration using UV-Vis (Nanodrop) (4.284 mg/mL)

Table S4. Experimental setup of spotting verification

**Strip configuration**
Backing card: Diagnostic Consulting Network, 60 mm<br>Conjugate pad: GFDX203000 (Millipore), dx=0 mm, length=23 mm<br>Nitrocellulose: Satorius CN95, dx=16 mm, length=25 mm (overlap=7 mm)<br>Wicking pad: CF5, dx=33 mm, length=22 mm (overlap=9 mm)
**Spotting**
1 µL in the middle of the uncovered nitrocellulose (dx=28 mm)<br>IgG-biotin solutions: 0.02, 0.1, 0.5 mg/mL in PBS<br>Done by the robot or by a human operator
**Running**
15 µL on conjugate pad, gold nanoparticle-streptavidin (Arista Biologicals, CGSTV-0600) , with OD 0.1 in PBST (PBS with Tween-20 0.05%)<br>60 µL of PBST<br>Run by the robot
**Imaging**
On the robot, when dried
**Number of technical replicates**
16, for each biotinylated IgG concentration and operator (robot or human)

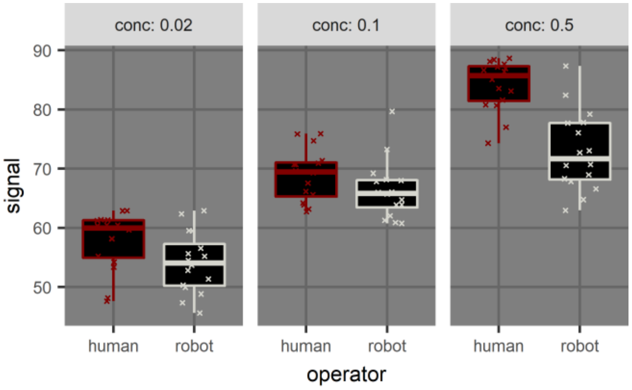

Figure S7. Plots showing signal of spotting verification, done by a human operator or a robot, with different concentrations biotinylated mouse IgG (0.02, 0.1, 0.5 mg/mL). Experimental details are described in Table S4 (N=16 for each box plot).

Table S5. Coefficients of variation (CVs) of spot intensities resulting from spotting by a human operator and the robot (N=16).

Concentration of capture reagent (mg/mL)	Human CV	Robot CV
**0.5**	5%	9%
**0.1**	6%	7%
**0.02**	8%	10%

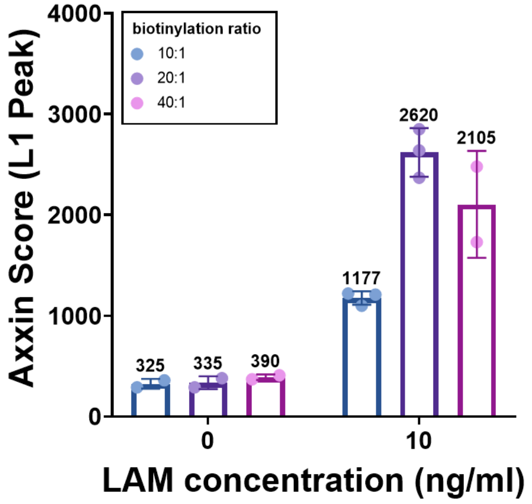

Figure S8. Comparison of biotinylated TBL005 when biotinylated at three different ratios; 10:1, 20:1, and 40:1. Antibody pair screening used 10:1 as the biotins/Ab ratio for screening to maintain consistency across all antibodies tested. However, this data shows that the optimal ratio for this particular antibody would be closer to 20:1. This suggests that selecting one ratio for all antibodies may bias towards or away from certain antibodies and is something that must be considered during experimental design.
